# Time to sputum culture conversion and its determinants among Multi-drug resistant Tuberculosis patients at public hospitals of the Amhara Regional State: A multicenter retrospective follow up study

**DOI:** 10.1371/journal.pone.0199320

**Published:** 2018-06-21

**Authors:** Temesgen Yihunie Akalu, Kindie Fentahun Muchie, Kassahun Alemu Gelaye

**Affiliations:** Department of Epidemiology and Biostatistics, Institute of Public Health, College of Medicine and Health Sciences, University of Gondar, Gondar, Ethiopia; University of Minnesota, UNITED STATES

## Abstract

**Background:**

In Ethiopia, Multi-drug resistant Tuberculosis (MDR-TB) is one of the major public health problems that need great attention. Time to sputum culture conversion is often used as an early predictive value for the final treatment outcome. Although guidelines for MDR-TB are frequently designed, medication freely provided, and centers for treatment duly expanded, studies on time to sputum culture conversion have been very limited in Ethiopia. This study was aimed at determining the time to sputum culture conversion and the determinants among MDR-TB patients at public Hospitals of the Amhara Regional State.

**Methods:**

A retrospective follow up study was conducted between September 2010 and December 2016. Three hundred ninety two MDR-TB patients were included in the study. Parametric frailty models were fitted and Cox Snell residual was used for goodness of fit, which the Akaike’s information criteria was used for model selection. Adjusted hazard ratio (AHR) with a 95% confidence interval (CI) was reported to show the strength of association.

**Result:**

Out of the 392 participants, sputum culture changed for 340(86.7%) during the follow up period. The median culture conversion time in this study was 65 (60–70 days). Alcohol drinking (AHR = 3.79, 95%CI = 1.65–8.68), sputum smear grading +2 (AHR = 0.39, 95%CI 0.19–0.79), smear grading +3 (AHR = 0.30, CI = 0.14–064), cavitations (AHR = 0.36, 95%CI = 0.19–0.68), and consolidation (AHR = 0.29, CI = 0.13–0.69) were the determinants of time to sputum culture conversion.

**Conclusion:**

In this study, time to sputum culture was rapid as compared to 4 months WHO recommendation. Alcohol drinking, sputum smear grading, cavitations and consolidations were found to be the determinants of time to sputum culture conversion. Therefore, providing a special attention to patients who had baseline radiological finding is recommended, high bacillary load and patients with a history of alcohol intake at baseline should be given priority.

## Introduction

Tuberculosis (TB) which kills approximately 2 million globally every year has been the second leading cause of morbidity next to the human immune deficiency virus (HIV) [1, 2]. The multidrug resistant (MDR) TB is an increasing global problem [3]. Proper treatment and enhanced case detection is needed to ensure good success rate and halt the emergency of MDR or extensively drug resistant (XDR) TB, as the risk is high among previously treated patients [4]. The treatment of MDR-TB takes 2 years (18–24 months), and monthly sputum culture is considered as a remarkable indicator of treatment effectiveness, especially during the intensive phase of treatment [5]. Knowing the time to sputum culture conversion is often used as an early predictive value for the final treatment outcome, especially in MDR-TB patients [2], and a delayed after four-month conversion was considered as a precondition for suspecting MDR-TB treatment failure [2].

According to 2017 global report, there were an estimated 600,000 (range, 540 000–660 000) new cases of MDR/Rifampin resistance (RR)-TB were reported. Of this, only 22% were officially diagnosed and 54% cure rate [6].

About 88% of the anticipated MDR-TB cases were found in Brazil, China, India, the Russian Federation, and South Africa [7], while its impact worsened in Asia and Africa [8]. According to the 2015 global report, 28% of MDR-TB patients were found in the African region [2], Ethiopia ranked 15^th^ among the 27 high MDR-TB burden countries with more than 5000 estimated MDR-TB patients each year. The prevalence is 2.8% in newly diagnosed patients and 21% in patients under re-treatment [9].

The most common factors that affect time to sputum culture conversion are: MDR-TB category, HIV co-infection, presence of radiological findings, presence of chronic diseases, number of resistant drugs at initiation, number of active drugs taken, and therapeutic delay of greater than one month [10–13].

Delaying sputum culture conversion resulted in economic wastage by prolonging the duration of treatment, poor treatment adherence and consequently failing treatment. It is also associated with higher case fatality rates (50–80%) as a result of drug toxicity, leading to the emergence of XDR-TB [13]. Despite the provision of free treatment, monthly culture monitoring, and expansion of services, studies on time to sputum culture conversion in our country including the study area is very limited. Therefore, this study is aimed to determine the time to sputum culture conversion and its determinants among MDR-TB patients.

## Materials and methods

### Study design and setting

An institution based retrospective follow up study was conducted from September 2010 to December 2016 among MDR-TB patients in three at the nine public hospitals of Amhara Regional State. Namely: University of Gondar Referral Hospital (UOGRH), Borumeda Hospital, and Debre-Markos Referral Hospital. These hospitals were among the nine hospitals in the region, at which about 80% of the patients received treatment.

University of Gondar Referral Hospital, which is located in North Gondar Administrative Zone, the Amhara Regional State, started MDR-TB treatment as a pilot program with the Global Health Commute (GHC) to treat patients as a national response to the emerging threat of drug resistant TB in September 2010. Currently, Gondar town has a referral hospital, eight health centers, and fourteen health posts owned by the government. There also are one general Hospital, thirteen specialties, fifteen medium, and ten primary clinics run by private sector. The University of Gondar Referral Hospital is a teaching hospital which serves more than five million people of North Gondar and neighboring zones, as well as the Tigray Region. The second setting is Debre-Markos Referral Hospital, which is located in Debre-Markos town (capital of East Gojjam). It is 297 km from Addis Ababa and 264 km from Bahirdar, the capital of the region. The MDR-TB center established recently was giving service to 50 patients. The third setting is Borumeda Hospital located 10 km from Dessie, the capital of South Wello and 441 km from Addis Ababa. The center was established in 2013 and was serving 131 MDR-TB patients.

### Population and sample

Gondar, Debre-Markos and Borumeda referral hospitals were selected out of the nine hospitals in the region. All drug resistant TB patients who were culture positive at the start of treatment at least for two months and had follow up were included in the study. Patients who had incomplete data on the outcome variables were excluded. Culture positive MDR-TB patients followed for less than two months and were culture negative at baseline were excluded. A total of 392 MDR-TB patients which fulfilled the inclusion criteria took part in study.

### Data collection procedures and variables

Time to sputum culture conversion was the response variable, where as socio-demographic characteristics (sex, age, residence, educational status, marital status), behavioral factors (smoking, alcohol drinking) and clinical characteristics(HIV co-infection, MDR category, MDR treatment regimen, type of resistance, presence of chronic diseases, clinical complications, radiological findings, therapeutic delays> 1 month, base line Body Mass Index (BMI), smear grading and functional status) were the explanatory variables.

Time to sputum culture conversion- was defined as the time from initiation of treatment of MDR-TB for a patient who had two negative consecutive cultures taken at least 30 days apart after initiation of treatment, censored was defined as when the outcome of interest has not been observed for an individual. This includes treatment stopped while culture was positive, died before conversion, transferred out before conversion, and study time completion before culture conversion. Therapeutic delay was defined as confirmed MDR-TB patient who started treatment after one month of MDR-TB diagnosis, previously not treated for TB was defined as a patient who had no prior anti-TB treatment or taken anti TB for less than one month. Previously treated case was defined as a patient who was treated for TB for one month or more, a patient who had under <18.5 kg/m2 body mass index was classified as low BMI, where as a patient who had ≥18.5kg/m2 body mass index was classified as normal BMI. Acid Fast Bacilli (AFB)Sputum smear microscopy was performed at baseline and on monthly follow-up using Ziehl- Nielsen staining. Results are reported based on the number of AFB: negative (no AFB/ 100 high-power fields [HPF]), scanty (1–9 AFB/100 HPF), 1+ (10–99 AFB/100 HPF), 2+ (1–9 AFB/HPF), and 3+ (>9 AFB/HPF).

### Data management and analysis

Prior to data collection, records (both baseline and follow up) were reviewed and identified by their medical registration/card numbers. Trained data collectors (nurse and health officer) reviewed and extracted data from patient charts and registries, using a semi structured check list. The filled formats were checked for completeness by the principal investigator and three supervisors. The data were entered in to EPI info version 7 and exported to Stata version 14 statistical software for further analysis.

Descriptive summary statistics, like the median time of culture conversion and Kaplan Meier curve estimations were computed. Proportional hazard assumption (PHA) was checked using graphs and Schoenfeld residuals tests. The Cox regression model for bivariable and multi variable analyses was done. Model comparison was carried out based on Akaike Information Criterion (AIC). Then, parametric frailty models were fitted and compared to identify the potential determinants. Goodness of fit was checked by Cox Snell residual test. Final results were taken as significant at 5% level of significance. The Adjusted Hazard Ratio (AHR) with its respective 95% Confidence Interval (CI) was reported to show the strength of association.

### Ethical issues

The ethics committee of Institute of Public Health, College of Medicine and Health Sciences, University of Gondar approved this study. Similarly, informed consent was not taken from the study participants directly as it is entirely secondary and no access to meet the study subjects. However, the committee gave accreditation for the approval and permission letter was obtained from Amhara regional state. Then permission was also requested from each hospitals and informed consent also waived by each respective hospitals for their patients. All data were de-identified prior to access by authors. The check list was kept securely in locked cabinets and the data base was password protected.

## Results

### Baseline socio-demographic characteristics

About 428 pulmonary MDR-TB patients were reviewed. Among these, 36 (8.4%) were excluded from the study because of baseline negative culture and contaminated results. Thus, a total of 392 (91.6%) study units were included in the analysis. The majority of the patients were attended at the University of Gondar Referral Hospital; 242(61.7%) were followed at Borumeda hospital,113(28.8%), and the remaining at Debre-Markos Referral Hospital.

The median age of the MDR-TB patients at initiation of treatment was 29.5 years IQR (20–40 years). One-third of the participants, 127 (32.4%), were under 25years of age. More than half of the respondents, 228(58.2%), were males and about half of them, 200(51%), were urban dwellers. About 168(42.9%) were private workers, and 314 (80.1%) were Orthodox Christian’s. Around 40% of the MDR-TB patients were not formally educated, and 164 (41.8%) were married (Table 1).

**Table 1:** Baseline socio demographic and behavioural characteristics of MDR-TB patients at initiation of MDR treatment in the Amhara Regional State public hospitals, September 2010 to December 2016 (n = 392).

Variables	Frequency	Percentage
Age		
<=24	127	32.4
25–34	126	32.2
35–44	73	18.6
>45	66	16.8
Sex		
Male	228	58.2
Female	164	41.8
Place of residence		
Urban	200	51.0
Rural	192	49.0
Occupational status		
Unemployed	89	22.7
Governmental	36	9.2
Private	168	42.9
Daily laborer	37	9.4
Student	40	10.2
House wife	22	5.6
Muslim	72	18.4
Other*	6	1.5
Religion		
Orthodox	314	80.1
Educational status		
No formally educated	156	39.8
Grade 1–8	12	31.6
Grade 9–12	74	18.9
Tertiary and above	38	9.7
Marital Status		
Married	199	50.7
Single	135	34.4
Divorced	50	12.8
Widowed	8	2.1
Baseline Smoking		
Yes	56	14.3
No	326	85.7
Baseline Alcohol drinking history		
Yes	77	19.6
No	312	80.4

*protestant and catholic

### Baseline clinical status

Among the 392 MDR-TB patients, 9.9% had chronic disease. Of these, 12 (3.1%) had diabetic mellitus (DM) and 4 (1%) hypertension (HTN). About one fourth (25.8%) of the MDR-TB patients had HIV co-infection, the majority 272(69.4%) were ambulatory, and 376 (95.9%) hospitalized at initiation of treatment. About 11 (2.8%) of the participants had previous MDR-TB treatment, and nearly half 199(50.8%)were mono resistant (Rifampin resistant). Regarding the x-ray finding, 107 (27.3%) of the MDR-TB patients had infiltration and 160(40.82%) cavitations. About 286 (73.0%) of the participants had low BMI (<18.5kg/m2) and 7 (1.8%) had treatment modification. Of 7 MDR-TB patients, 5 were MDR-TB treatment failure suspect, one patient was XDR-TB confirmed, and one was a life threatening drug side effect. About350 (89.3%) were previously treated for TB (Table 2).

**Table 2:** Baseline clinical statuses of MDR-TB patients at initiation of treatment in the Amhara Regional State public hospitals, September 2010 to December 2016.

Clinical factors	Frequency	Percentage
HIV co-infection		
Yes	101	25.8
No	291	74.2
Chronic disease		
Yes	39	9.9
No	353	90.1
Functional status		
Working	47	12.0
Ambulatory	272	69.4
Bedridden	73	18.6
Model of Rx initiation		
Hospitalized	376	95.9
Ambulatory	16	4.1
Type of resistance		
Mono resistance	199	50.8
MDR &XD	193	49.2
BMI		
<18.5 kg/m2	286	73.0
>=18.5	106	27.0
Rx modified		
**Y**es	7	1.8
No	385	98.2
Registration group		
Previously not treated for TB	42	10.7
Previously treated for TB	350	89.3
Cavitations		
Yes	232	59.2
No	160	40.8
Infiltrations		
Yes	285	72.7
No	107	27.3
Consolidation		
Yes	344	87.8
No	48	12.2
Chronic Fibrotic change		
Yes	305	77.8
No	87	22.2

### Diagnosis method and treatment outcome

**Fig 1:**
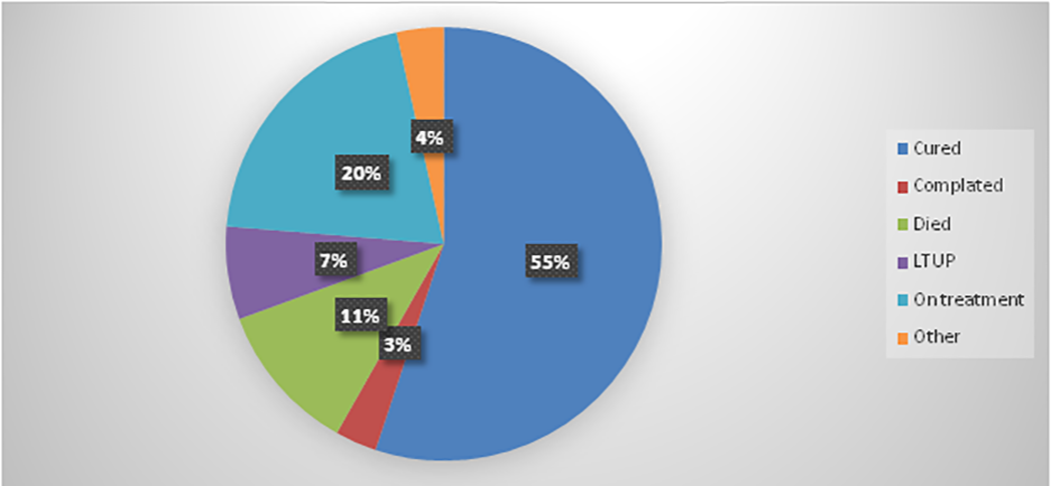
Overall treatment outcome of MDR-TB patients at public hospitals in the Amhara Regional State, September 2010 to December 2016.

Next to cough, fever, which affected 366 (93.4%) was the most common presentation of the patients, followed by 365 (93.1%) who lost weight. Of 392 MDR-TB patients, 136 (34.7%) had shortness of breath and 285 (72%) chest pain. About 319 (81.4%) participants had history of fatigability and 305 (77.8%) history of poor appetite. The methods of diagnosis employed for 177 (45.2%), 139 (35.4%), 64 (16.3%), and 12 (3.1%) of the MDR-TB patients were Mycobacterium Tuberculosis (MTB) /gene expert, Line Probe Assay (LPA), culture/Drug susceptibility test (DST), and clinical, respectively. Of the 392 MDR-TB patients, 52 (13.3%) were censored and 340 (86.7%) converted their culture. The overall cure rate of MDR-TB patients was 216(55.1%) and 12 (3.1%) completed their treatments (Fig 1).

### Time to sputum culture conversion

**Fig 2:**
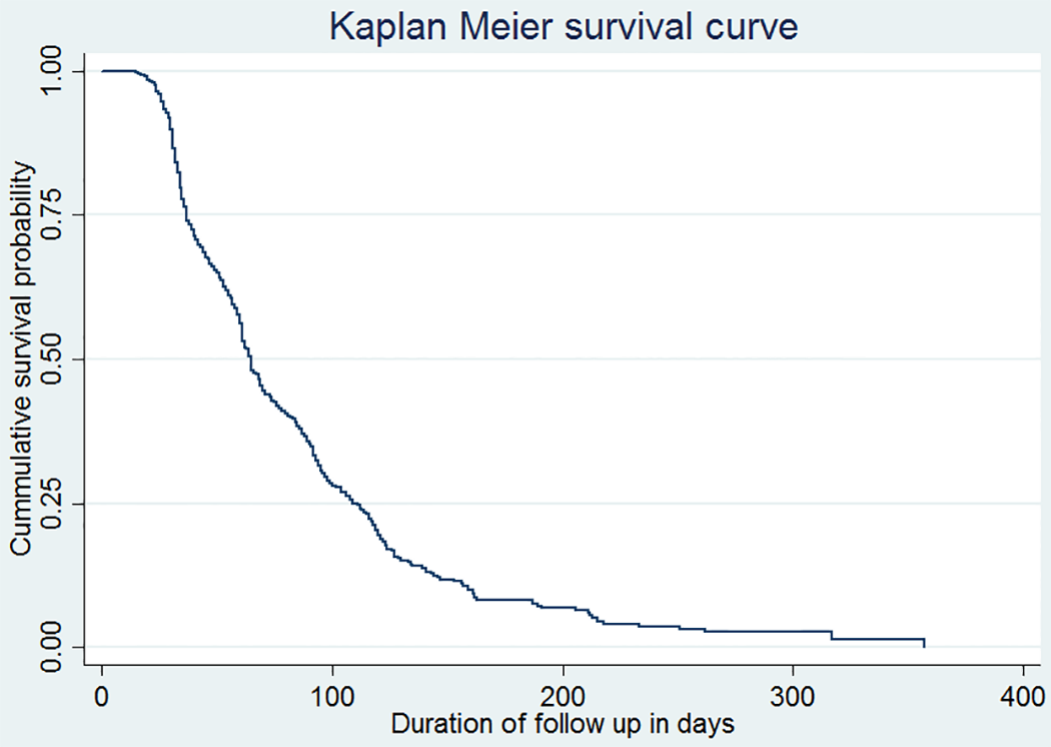
Kaplan-Meier survival curve of MDR-TB patients on initial culture conversion at public hospitals in the Amhara Regional State, September 2010 to December 2016. Differences in all variables at baseline between strata were determined using the log rank (χ2) test, and the equality of hazard was assessed for the different explanatory variables. According to the test results, the presence of infiltration p-value = 0.00, cavitations p-value = 0.00, consolidation p-value = 0.01, Fibrotic change p-value = 0.00, functional status p-value = 0.03, sputum smear grading p-value = 0.00 and resistance type p-value = 0.00associated with initial culture conversion time at 5% level of significance.

The participants were followed for a total of 1000.6 person months (83.4 person years) observation. The median follow up period was 65 days (IQR: 61–70). The cumulative probability of survival on initial conversion was 0.89 at the end of one month, 0.56 at the end of two months, and 0.19 at the end of four months (Fig 2).

**Fig 3:**
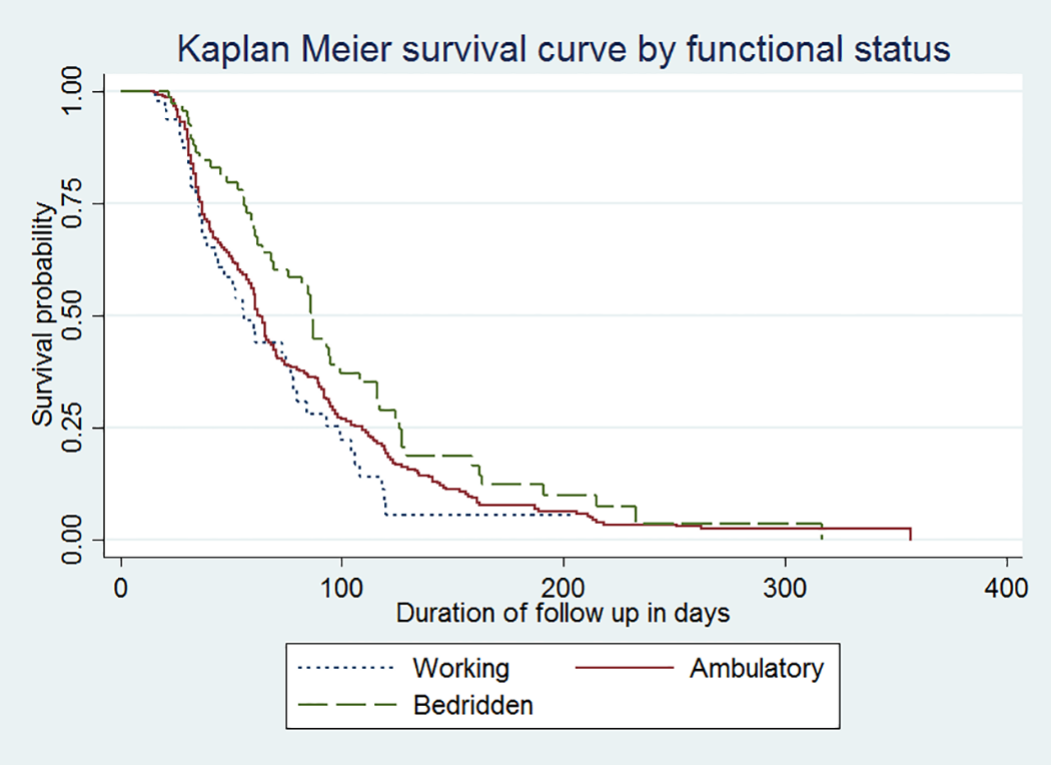
Kaplan-Meier curve of surviving MDR-TB patients on initial culture conversion by baseline functional status at public hospitals in the Amhara Regional State, Ethiopia, September 2010 to December 2016.

The Schoenfeld global test of the functional status satisfies the PHA (P-value = 0.294). The mean survival time, on culture conversion for patients who were bedridden had a median conversion time of 87 days, whereas ambulatory and working patients had median conversion times of 63 and 56, respectively, and the difference was significant (p-value = 0.0318) (Fig 3).

**Fig 4:**
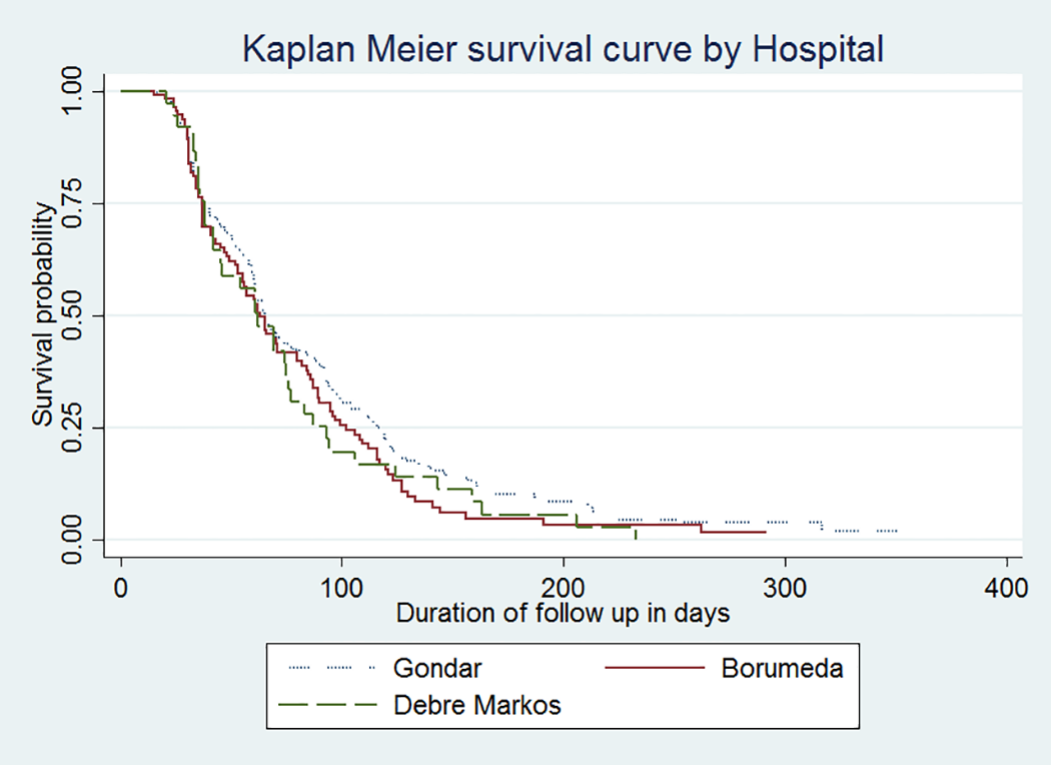
Plot of the probability of survival by hospital and the time of follow up (in days) among MDR-TB patients at public hospitals in the Amhara Regional State, September 2010 to December 2016.

The median culture conversion times at Gondar, Borumeda, and Debre-Markos hospitals were 65 CI (61–76), 63 (53–80), and 62 (42–76) days, respectively (Fig 4).

### Determinants of time to sputum culture conversion

**Fig 5:**
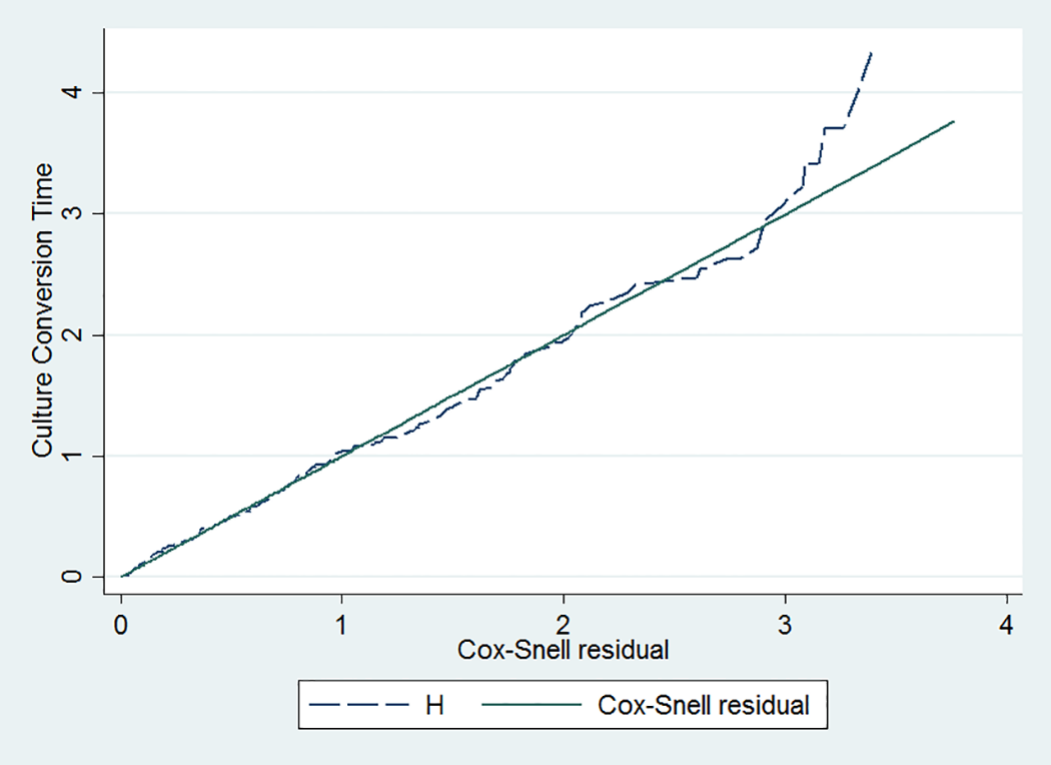
Goodness of fit test for univariate Weibull distribution gamma frailty model.

Similarly, the proportional hazard assumption was checked by the Schoenfeld residual global test, and p-value showed 0.68. So, proportional hazard assumption was met. Based on Akaike Information Criterion (AIC), the univariate frailty with Weibull distribution and gamma frailty (AIC = 396.82) model was more efficient than Cox-proportional hazard (AIC = 1859.41), parametric exponential model (AIC = 545.05), and other frailty models (Table 3). Thus, the inclusion of shared frailty term showed no statistically significant variance and indicated that there was no any shared heterogeneity or shared unobserved variability among hospitals. But there was a statistically significant heterogeneity among individuals (variance = 1.56)(Table 4). Goodness of fit for the fitted model was also performed using the Cox Snell residual test and showed that the model was adequate (Fig 5).

**Table 3:** Model comparison criteria based on AIC and Log likelihood test for different survival models.

Model	Baseline hazard	Frailty	Variance	Log-likelihood	AIC
Cox	Unspecified			-919.71	1859.41
Exponential Reg	Exponential			-261.89	545.05
Weibull Reg	Weibull			-200.99	425.99
Gompertz Reg	Gompertz			-237.33	498.66
Univariate frailty	Weibull	Gamma	1.56(P<0.001)	-185.41	396.82
Univariate frailty	Weibull	Inversegaussian	3.1(P<0.001)	-189.53	405.06

**Table 4:** Univariate Weibull distribution gamma frailty model for determinants of time to sputum culture conversion among MDR TB patients in the Amhara Region State public hospitals, September 2010 to December 2016.

Variable	Censored	Event	CHR 95%CI	AHR 95%CI
**Baseline Alcohol history**				
Yes	14	63	1	1
No	38	277	2.42(1.83,3.21)	3.79(1.65–8.7)*
**Type of resistance**				
Mono resistance	34	165	1	1
MDR and XDR	18	175	0.72(0.58,0.89)	0.73(0.42,1.28)
**Sputum smear grading**				
Smear G-1	4	114	1	1
Smear G-2	9	53	0.65(0.47,0.89)	0.39(0.19,0.79)*
Smear G-3	12	44	0.37(0.26,0.54)	0.30(0.14,0.64)*
**Cavitations**				
No	24	208	1	1
Yes	28	132	0.52(0.41,0.64)	0.36(0.19,0.68)*
**Infiltration**				
No	32	253	1	1
Yes	20	87	0.55(0.43,0.71)	0.76(0.38,1.53)
**Consolidation**				
No	48	296	1	1
Yes	4	44	0.57(0.42,0.79)	0.29(0.13,0.69)*
**Chronic fibrotic change**				
No	40	265	1	1
Yes	12	75	0.63(0.48,0.82)	0.54(0.28,1.04)
**Functional status**				
Working	8	39	1	1
Ambulatory	23	249	0.8(0.5,1.1)	0.75(0.33–1.69)
Bedridden	21	52	0.5(0.4,0.8)	0.35(0.12,1.05)

*indicates p-value <0.05

The finding from the bivariate analysis showed that alcohol drinking, type of resistance, baseline sputum smear grading, and cavitations at baseline, baseline infiltration, baseline consolidation, chronic fibrotic change at the baseline, and baseline functional status were significantly associated with culture conversion time. However, in the multi-variable analysis, alcohol drinking status, baseline sputum smear grading, baseline cavitations, and consolidation remained statistically significant predictors of time to sputum culture conversion. The rate of sputum culture conversion among who didn’t drink alcohol at baseline was 3.8 times as compared to alcohol drinkers**(AHR = 3.8; 95%CI:1.7–8.7**).

The rate of sputum culture conversion among MDR-TB patients whose baseline sputum smear grading was +2 decreased by 61%(**AHR = 0.4;95%CI: 0.2, 0.8**) as compared to MDR-TB patients whose baseline smear grading was +1. Similarly, the rate of culture conversion among MDR-TB patients whose baseline smear grading was +3 decreased by 70% (**AHR = 0.3;95%CI 0.1, 0.6))** as compared with those baseline sputum smear was +1. Regarding the x-ray findings of MDR-TB patients who had cavitations at baseline decreased culture conversion time by 64% as compared to MDR-TB patients without cavitations**(AHR = 0.36;95%CI:0.19,0.68)).** Similarly, the rate of sputum culture conversion among MDR-TB patients who had consolidation at baseline decreased conversion time by 71% as compared to MDR-TB patients who had no consolidation (**AHR = 0.29;95% CI:0.13,0.69))** (Table 4).

## Discussion

In this study the median culture conversion time was 65 days. Previous baseline alcohol drinking status, baseline sputum smear grading, radiological findings, like cavity and consolidation were found to be the determinants of time to sputum culture conversion.

The median culture conversion time found in this study is in line with that of studies conducted in South Africa [14] and Georgia [15]. However, this finding showed a delayed conversion compared with a study conducted in Peru [16], which has a median conversion time of 59 days. In Peru [16]these DST results as well as other prior anti-TB drug exposures were taken into account when formulating the treatment regimen, but in our study DST was done when the start of treatment had some indication like previous history of MDR-TB, patients with HIV AIDS, failure with first line anti-TB, and contact with known MDR-TB treatment. In contrast, our study showed a rapid culture conversion time compared with multi centered studies conducted in five countries such as Peru, Latvia, Estonia, Russia, and the Philippines in which the median culture conversion time was 3 months (90days). Similarly, a study in Pakistan had a median conversion time of 191 days [13, 17]. This might be due to the way outcome variables were defined. In our study culture conversion time was explained by two consecutive negative culture results, whereas this was done by five consecutive negative culture results in the multicenter study. The second possible reason might be that a very small sample was used in the Pakistan study (N = 85). The third reason could be the fact that in present study MTB/Rif Xpert was used upon a positive sputum smear microscopy and the detection of Rifampicin resistance to place on standardized treatment regimen right from the beginning rather than waiting for DST results [17].

In MDR-TB patient’s, alcohol drinking resulted in delayed culture conversion time. This finding is in line with that of a multi centered study conducted in five countries like Peru, Estonia, the Philippines, Latvia, and Russia [13]. This might because a large number of MDR-TB patients who drink alcohol had cavitations in the multicenter study [13]. The second reason could be related to poor nutritional status, and the direct toxic effects of ethanol on the immune system or poorer adherence to anti-tuberculosis treatment [18, 19]. The third reason might be that alcoholism causes drug resistance by decreasing the immunity status [20].

In MDR-TB patients smear +2 and +3 resulted in delayed culture conversion time. This finding is in line with that of a study conducted in Latvia [21], Korea [22], and Indonesia [23]. Patients with high smear grading had high bacillary load. Having a high bacillary burden suggests a stronger infectivity and requires a longer isolation period and a more intensive treatment. Thus, it needs time to clear the bacilli if the bacillary load is high [22]. The second possible explanation could be that the presence of a high bacillary load is better associated with a reduction in bacterial killing and sterilizing activity of anti-TB drugs. It may be natural that patients with higher colony counts take longer time to convert sputum cultures [24].

Multi drug resistance TB patients who had baseline cavitations had delayed conversion time. The negative association between time to sputum culture conversion and lung cavitations in our study is in line with those of studies conducted in China [25] and Pakistan [26]. The possible reason might be the presence of lung cavitations decrease penetration and anti-bacterial activity of drugs [27]. In contrast to our finding, studies conducted in South Africa found no significant association between lung cavitations at baseline chest X-ray and culture conversion time [28]. The difference might be explained by a larger sample size and the four fold lower proportion of HIV co-infected patients used in our study [28].

The presence of baseline consolidation on chest radiography was associated with a longer time to initial sputum culture conversion as compared with MDR-TB patients who had no baseline consolidation. Consolidation occurs through the accumulation of inflammatory cellular exudates in the alveoli and the adjoining ducts. The liquid can be pulmonary edema, inflammatory exudates, pus, inhaled water, or blood (from bronchial tree or hemorrhage from a pulmonary artery). This minimizes the response to MDR-TB treatment and may delay the culture conversion time [29]. In contrast to this finding, a study conducted in South Africa revealed that there was no significant relationship between baseline consolidation and time to sputum culture conversion. This difference might be explained by the use of a small sample (n = 56) and a large number (88%) of HIV positive participants used in the study conducted in South Africa, showing these groups were immune-compromised, since, a radiological finding in immune compromised persons like HIV may not have sufficient lung finding [28].

Sputum culture conversion in MDR-TB patients is an important routine indicator in monitoring treatment outcome. Achieving more rapid sputum culture conversion can increase patients comfort by reducing the duration of injectable drug use and simplifying a patient’s therapy. In addition, from the public health perspective, reducing the time to sputum culture conversion is an important infection control measure because patients with MDR-TB and positive sputum cultures are infectious and may transmit the disease to other persons, family members, and health care providers [21]. The strength of this study is its incorporation of a multi-center site, and implementation of the frailty model estimation instead of a binary end point (logistic regression) for addressing the effect of unobserved heterogeneity, and handling interval censoring by using parametric survival analysis. Since the data were collected from secondary source, some important predictors like HGB, BCG scar, and income which had a significant association with time to sputum culture conversion in other studies were missed.

## Conclusion

The time to culture conversion in our study is rapid as compared to WHO guidelines which recommend four months. Previous alcohol drinking, high baseline sputum smear grading, presence of cavitations and consolidation were found to delay time to sputum culture conversion.

Close follow-up and attention is important especially for those who were alcohol drinkers, have baseline cavitations, and consolidation. It is also useful to assess baseline sputum smear grading and provide great attention to those who have had a high bacillary load. Finally, we recommend that researchers conduct prospective follow up studies which include other independent variables (Anaemia, BCG scar, income).

## Supporting information

S1 Checklist (DOCX)

## Abbreviations

AFB: Acid fast bacilli
AIC: Akaike Information Criteria
BIC: Bayesian information Criteria
BMI: Body Mass Index
CHR: Chest X-RAY
CHR: Crude Hazard Ratio
CS: Cyclo Serine
DM: Diabetic Mellitus
DOTS: Direct Observed Therapy Short Course
ETHO: Ethionamid
HGB: Hemoglobin
HIV: Human Immune- Deficiency Virus
HPF: High Power Field
HR: Hazard Ratio
HTN: Hypertension
Kg/m^2^: Kilogram Per Meter Square
LFX: Levofloxacin
LPA: Line Prove Assay
MDR: Multi Drug Resistant
PAS: Paramino Salicylic Acid
PI: Principal Investigator
PRO: Prothionamide
TB: Tuberculosis
TIC: Treatment Initiating Center
UOGRH: University Of Gondar Referral Hospital
WHO: World Health Organization
XDR: Extensive Drug Resistant
Z: Pyrazinamide
