# Supplementary material for: Time to sputum culture conversion and its determinants among Multi-drug resistant Tuberculosis patients at public hospitals of the Amhara Regional State: A multicenter retrospective follow up study
Source: PLoS One. 2018 Jun 21;13(6):e0199320. doi: 10.1371/journal.pone.0199320 (PMC6013102; doi:10.1371/journal.pone.0199320)
Supplement: S1 Checklist — (DOCX) [file pone.0199320.s001.docx]

# **Annex**

## **Annex I Data collection Check list**

This checklist is prepared for the collection of socio-demographic, clinical, behavioral factors, and treatment outcome related information that are important for the assessment of outcome and predictors of time to sputum culture conversion in University of Gondar Hospital, Debre Markos hospital and Borumeda hospital. All these information were retrieved from the clients MDR-TB registration book and from individual patient card without mentioning the name of clients. This information was collected by health care providers (BSc nurse or Health Officer).

**Contact Information: *Temesgen Yihunie Tel +251-929-390-709 Dr. Kassahun Alemu Tel +251-911-752-466 Mr. Kindie Fentahun*** [***Tel:***](Tel:+251-912-180307)**+251928436726**

| s.n | Variables | Labels  . |
| --- | --- | --- |
| 1 | Patient identification code | Unique code------------- |
| 2 | Name of hospitals | 1.Gondar--------------  2.Borumeda-----------  3.D/markos------------ |
| 3 | Age of patient in years | ______years |
| 4 | Sex | 1. Male 2. Female |
| 5 | Address | Region----- zone------woreda-------kebele---- |
| 6 | Place of Residence | 1.Urban 2.Rural |
| 7 | Occupation | 1. Unemployment 2.Goverment 3.None government 4.Private 5. Other-------- |
| 8 | Religion | 1. Orthodox 2.muslim 3.Protestant 4.Catholic 5. Other- |
| 9 | Baseline weight | ___ in Kg |
| 10 | Baseline height | -------in centimeter |
| 11 | Educational status | 1. No educated 2. Primary 3. Secondary 4. Tertiary |
| 12 | Marital status | 1. Married 2. Never married 3. Divorced 4. Widowed 5. Other------ |
| 13 | Baseline smoking | 1. Yes 2. No |
| 14 | Baseline alcohol drinking | 1. Yes 2. No |
| 15 | Presence of other chronic diseases | 1. Yes 2. No 3. Unknown |
| 16 | If yes for Q 15then what are? | 1. DM 2.HTN 3. CKD 4.Bronchial asthma  5. Other(specify) |
| 17 | Registration group | 1. New 2. Relapse 3. After LTFP 4. After failure of first line treatment 5. After failure of retreatment 6. Other |
| 18 | Previous MDR TB treatment | 1. Yes 2. No |
| 19 | Model of treatment initiation | 1. Hospitalized 2. Ambulatory |
| 20 | Date of MDR-TB Diagnosis | dd/mm/yy ----/------/------ |
| 21 | Date of MDR-TB treatment started | dd/mm/yy ----/------/------ |
| 22 | Time of sputum culture converted | dd/mm/yy----/----/---- |
| 23 | functional status at first admission | 1. Working 2. Ambulatory 3. Bedridden |
| 24 | Type of resistance | 1.mono resistance 2.poly resistance 3.MDR 4.XDR |
| 25 | To which anti TB drug patients resistance | 1.R only 2.R& H 3.R,H,E,S |
| 26 | Laboratory Diagnosis confirmed by | 1.MTB/RIF gene Xpert 2.LPA  3.conventionl DST |
| 27 | Any TB related complication | 1. Yes 2. No |
| 28 | If yes to Q.25 type of complication | 1. Pneumothorax 2. Pneumonia 3. Hemoptysis 4. Cor pulmonale 2. Other----------- |
| 29 | HIV co-infection | 1. No 2. Yes |
| 30 | MDRTB Treatment regimen | 1. cs-z-etho/pto-lfx-cm  2. cs-z-PAS-lfx-cs 3.other----------------------- |
| 31 | Radiological finding | 1. Cavity 2. Infiltration 3. Consolidation 4. Effusion 5. Hilar LAP 6. Fibrotic change 7. Other |
| 32 | Baseline sputum smear grading | 3+(>9 AFB/HPF)  2+(1-9 AFB/HPF)  1+(10-99AFB/100/HPF)  Scanty (1-9 AFB/ HPF)  Negative |
| 33 | Final treatment outcome | 1. Cured  2.completed  3.died  4.LTF  5. transferred out  6. On treatment |
| 34 | Symptoms at admission | 1. Shortness of breath 2. Fever 3. Weight lose 4. Hemoptysis 5. Chest pain 6. Sweating 7. Fatigability 8. Other |

Collected

by____________________Signature ____________Date ________________

Supervised

by___________________Signature ____________ Date _______________
